# Codeveloping a Novel Intervention to Promote the Well-Being of Family Caregivers of Individuals With Spinal Cord Injury: Protocol for a Feasibility Randomized Control Trial

**DOI:** 10.2196/67709

**Published:** 2025-09-25

**Authors:** Somayyeh Mohammadi, Beth Erlander, Heather Cathcart, Julie M Robillard, Sophia Sauvageau, David G T Whitehurst, Elena Pauly, Brooke Pagé, William C Miller

**Affiliations:** 1 Department of Occupational Science and Occupational Therapy University of British Columbia Vancouver, BC Canada; 2 Rehabilitation Research Program, Centre for Aging SMART, GF Strong Rehabilitation Centre Vancouver, BC Canada; 3 Grief Coaching and Counselling Services Boulder, CO United States; 4 BC Children’s & Women’s Hospitals Vancouver, BC Canada; 5 Division of Neurology, Department of Medicine, University of British Columbia Vancouver, BC Canada; 6 International Collaboration On Repair Discoveries Vancouver, BC Canada; 7 Faculty of Health Sciences Simon Fraser University Burnaby, BC Canada; 8 Wives and Girlfriends of Spinal Cord Injury Vancouver, BC Canada

**Keywords:** caregiver, eHealth, burden, spinal cord, spinal cord injury, chronic health conditions, mHealth, mobile health, family

## Abstract

**Background:**

Family caregivers of individuals with spinal cord injury (fcSCI) provide assistance with activities of daily living for individuals with spinal cord injury (SCI), which can include emotional support and physical assistance. Over time, providing daily support can put fcSCI at risk of experiencing caregiver burden. This burden can have a substantial impact on fcSCI’s well-being. A direct predictor of the fcSCI burden is their appraisal of their ability to cope with the individual with SCI. Therefore, supporting fcSCI’s access to education relevant to their role and the health and well-being of the individual with SCI can help decrease this burden.

**Objective:**

The purpose of this study is to evaluate the fidelity of the COMPANION intervention and its study protocol designed to improve outcomes for fcSCI.

**Methods:**

Recruitment began in June 2024 and is ongoing. Data collection was completed in April 2025. The fcSCI randomized into the intervention group have been given access to COMPANION after randomization. Data collection for both participant groups (COMPANION and control) will be performed after randomization and will be performed again after 3 months (T2) and 6 months (T3) to capture the impact of COMPANION on fcSCI’s psychological well-being.

**Results:**

As of June 2024, recruitment has begun and is ongoing. Data collection has begun and will conclude in 2025. The fcSCI randomized into the intervention group have been given access to COMPANION after randomization. Data collection for both participant groups (COMPANION and control) is collected after randomization, and again after three months (T2) and six months (T3) to capture the impact of COMPANION on fcSCI’s psychological wellbeing.

**Conclusions:**

Study results will evaluate whether the full study can and should be conducted and will lead to the refinement of COMPANION.

**Trial Registration:**

ClinicalTrials.gov NCT06364813; https://clinicaltrials.gov/study/NCT06364813

**International Registered Report Identifier (IRRID):**

DERR1-10.2196/67709

## Introduction

### Background

Approximately 360,000 individuals have spinal cord injury (SCI) in the United States [1] and a further 86,000 in Canada [2]. SCI causes substantial disruption in the patient’s life habits, routines, and level of independence. In many cases, individuals with SCI need considerable human assistance to perform any activities that exceed their motor and sensory capability [3]. Family caregivers (defined as “any relative, partner, friend or neighbor who has a significant personal relationship with, and provides a broad range of assistance for, an older person or an adult with a chronic or disabling condition” [4]) of individuals with SCI (fcSCI) often provide the majority of physical care to their family members with SCI [5]. Furthermore, individuals with SCI may experience higher levels of depression and anxiety and lower levels of life satisfaction than the general population [6]; therefore, fcSCI may also provide emotional support, alongside physical support. Providing physical and emotional care and support can have a substantial impact on fcSCI’s physical and psychological well-being [5]. Many fcSCI report physical, emotional, and financial challenges associated with their caregiving roles and tend to neglect their own mental and physical health [7-9]. Over time, these experiences put fcSCI at high risk of burnout from an increased caregiver burden, which is associated with decreased well-being [5,8].

Caregiver burden is defined as “the extent to which caregivers perceive that caregiving has had an adverse effect on their emotional, social, financial, physical or spiritual functioning” [10]. Previous research indicates that 40%-46% of fcSCI report moderate-to-severe levels of caregiver burden due to the long-term nature of the injury and the caregiver role [10]. The burden associated with caregiving also influences fcSCI’s ability to respond to the needs of individuals with SCI, and the long-term impact of caregiving demands on fcSCI may result in decreased support and quality of care for individuals with SCI. Hence, attending to risk factors and interventions that target the well-being of fcSCI is crucial.

Although family caregivers of individuals with other chronic conditions, especially dementia, receive considerable attention in research [11-13], the development and evaluation of interventions that address the unique challenges faced by fcSCI have received less attention. The informal caregiving integrative model [14] describes several factors that determine caregiver burnout, which can be divided into 3 main categories: (1) caregivers, that is, caregivers’ sociodemographic characteristics, psychological factors (eg, emotion regulation, perceived competence), and physical state; (2) caregiving setting, that is, primary stressors (eg, living with the care recipient, being a spouse) and secondary stressors (eg, having a reduced social life, loss of friends); and (3) social environment, that is, informal and partner support, professional support (eg, availability of professional support and relationship with health care providers), and sociocultural environment (eg, how the caregiving role is perceived in society). Although some of the factors impacting the caregiver burden cannot be modified (eg, gender or relationship), other factors, such as emotion regulation or relationships with health care providers, are modifiable. Specifically, the informal caregiving integrative model [14] explains that fcSCI’s appraisal of their ability to cope with individuals with SCI is a direct predictor of their burden. The fcSCI’s coping appraisal is directly associated with their need for available resources and knowledge regarding the health condition of individuals with SCI. Therefore, helping fcSCI access education can increase their appraisal of their coping ability and lower levels of the caregiver burden.

A recent scoping review by McKay et al [15] suggested that fcSCI maintain better well-being if they have enough leisure activities, strong problem-solving skills, and practical and emotional support. In a recent qualitative study of Canadian fcSCI, Jeyathevan et al [16] identified that access to community and social support, a better relationship with individuals with SCI, and mastery in caregiving could help fcSCI provide sustainable care for individuals with SCI and for themselves. Although the findings of Jeyathevan et al [16] identified several critical needs of fcSCI, few studies have investigated the impact of different types of interventions on outcomes for family caregivers’; the studies that have been conducted have focused mainly on teaching problem-solving skills [16-20]. Additionally, even though rehabilitation facilities invite fcSCI to join educational sessions, these sessions primarily focus on patients’ needs. Compounding this challenge, fcSCI often have limited time, making attendance at in-person sessions difficult.

### Objectives

In our objective to improve outcomes for fcSCI, this study will evaluate the implementation fidelity of an innovative intervention program for health education with an online approach (eHealth) that aims to improve the well-being of fcSCI. COMPANION, cocreated with caregiver partners and health care professionals, is an interactive eHealth education program that is based on adult learning principles [21] and a user-centered approach [22]. The goal of COMPANION is to address fcSCI’s specific challenges in caring for themselves and for individuals with SCI and facilitate the provision of accessible online education. We envision COMPANION will improve access to existing knowledge for fcSCI and individuals with SCI and provide customized (eg, developed through understanding caregivers’ perspective), interactive (eg, quizzes), and engaging eHealth education (eg, new educational videos and audios) in the form of online modules, which are essential aspects of an eHealth tool [23], to better prepare and support fcSCI for providing care for themselves and individuals with SCI.

## Methods

### Study Design

This paper provides the details of a study protocol for a concurrent mixed methods study, including a feasibility randomized controlled trial (RCT). The study is registered with ClinicalTrials (NCT06364813). The purpose of the feasibility RCT is to (1) assess the fidelity of the process, resource, management, and treatment indicators and (2) estimate the parameters needed for a large-scale, multisite RCT [24]. Secondary objectives will be to assess the effect of COMPANION on primary and secondary clinical outcomes for fcSCI [24,25].

To assess the feasibility and intervention fidelity of COMPANION, a 6-month feasibility RCT with a 1:1 allocation ratio will be conducted, comparing COMPANION with the standard education and resources available to fcSCI. The study will incorporate quantitative and qualitative design methods administered through surveys and a final exit interview. In conjunction with RCTs in rehabilitation, the use of qualitative methods has been recommended to spot unexpected variables and findings [26,27]. The interview will be an opportunity for participants to share perspectives about their experience using COMPANION, with findings used to interpret clinical outcomes and, when integrated with quantitative findings, identify patterns and paradoxes between the results.

### Ethical Considerations

The University of British Columbia Research Ethics Board has approved this study (approval number H20-01461). All participants will sign the informed consent form prior to responding to baseline measures. To protect participants’ identity, all identifying materials will be removed from the data files. This includes Qualtrics information, such as IP addresses and participant contact information. A master list containing the participants’ names will be created, and each participant will be given a unique study number. The study numbers will be used to identify participants. The master list will be kept separate, password-protected, encrypted, and filed away from the participant data. Consent forms will be kept in a separate password-protected folder. The semistructured interviews conducted 6 months after baseline (T3) will be transcribed verbatim, and identifiable information will be removed prior to beginning analysis. All participants will receive CA $50 (US $37) after completing each timepoint.

### Study Timeline

The following 3 data collection timepoints will be used in the feasibility RCT: the time immediately following successful screening of the participants, during which baseline data will be collected and access to COMPANION will be provided to participants in the intervention group; 3 months after baseline data collection (T2); and 6 months after baseline data collection (T3). Please see Multimedia Appendix 1 for further information about the study timeline.

As of June 2024, recruitment and data collection for the study began and are ongoing. All data collection is performed via a secure online survey tool available through our institute (Qualtrics). After successful screening, randomization, and baseline data collection have been conducted, participants will be asked to complete the baseline survey. Follow-up data will be collected at 3 months (T2) and 6 months (T3). At baseline-T3, participants will respond to measures that assess primary (subjective burden) and secondary (objective burden, relationship quality satisfaction, distress, physical and mental health, and caregiving competence) outcomes. The purpose of baseline is to collect baseline information about the outcomes of the study. The purpose of T2 is to capture the influence of the intervention on our psychological outcomes of interest (eg, depression, anxiety, stress, and competence). T3 reflects the optimal change period for self-reporting of physical outcomes (eg, physical health and functioning).

### Sample Size and Inclusion and Exclusion Criteria

Suggested sample sizes for a feasibility RCT range from 12 to 30 per experimental group [28,29]. We aim to recruit at least 20 participants per group. Inclusion criteria for participants are, at the time of recruitment, as follows:

- Family caregiver of an adult individual with SCI (similar to previous studies of this population [30,31]; in this study,
  family caregiver
  is defined as an individual who is primarily responsible for providing immediate informal daily care for a relative with SCI).
- Age >18 years.
- Living with the individual with SCI in the community.
- Living in North America.
- SCI of the patient has not happened within the previous 6 months.

Those fcSCI who had major medical and physical conditions that require routine visits to medical doctors (eg, cancer) will be excluded from the study, in addition to fcSCI providing care to individuals with SCI who are still patients in a rehabilitation facility.

### Recruitment

The fcSCI are approached to participate (1) indirectly by sending letter and email invitations to previous patients or research participants of our center (GF Strong Rehabilitation Center and the International Collaboration on Repair Discoveries [ICORD]) and asking them to invite their family caregivers to contact us and (2) directly by asking our caregiver partners, Wives and Girlfriends of Spinal Cord Injury, to approach their members (approximately 6000 members mainly in North America) and invite them to participate in this study. We used social media advertisements to facilitate recruitment. Participants are recruited internationally from locations across North America. To include the participation of individuals from remote and rural areas, all participants are invited to participate digitally through a virtual meeting platform. In-person participation is not offered regardless of physical proximity to the research team.

### Randomization and Masking

Upon successful enrollment and baseline data collection, participants are randomized by the research staff using the online service Sealed Envelope [32] with a block size that is undisclosed to the study manager. The research staff is responsible for randomization and the delivery of the intervention, while the study manager, who is responsible for enrolling participants and conducting data collection, remains masked to the participants’ group allocation. To mitigate performance bias, participants are instructed not to discuss their program or group allocation with any member of the research team, except the research staff, and both the study manager and research staff will continue to reinforce this point when contacting participants. The research staff is responsible for receiving emails from participants and removing information related to group allocation before forwarding participant emails to the study manager.

### Intervention

COMPANION was designed through a collaboration between researchers, health care providers, and family caregivers (end users and stakeholders) using the Technology Co-Design Model [33,34]. The current preliminary version of COMPANION consists of 8 self-paced online modules, which can be accessed remotely from any location.

### Intervention Group

Participants in the intervention group are able to choose the order in which they complete the COMPANION modules and are encouraged to complete them all. They are also encouraged to invite individuals with SCI to view the COMPANION materials with them, as several of the modules include exercises that the participants can do with individuals with SCI and information regarding processes that the fcSCI can navigate with individuals with SCI (eg, changes in their relationship, considerations for hiring a home care aide, and accessing financial and legal support). Participants receive the web address, instructions to access the website using personalized encrypted login information, and guidelines on how to use the program. Modules have been designed so they can be stopped at any point, with progress automatically saved. Module formats include embedded video clips, voiceovers, and additional resources, including links to other available information. As topics are introduced in the course, participants are reminded that the content in the modules is not a substitution for expert professional advice and are instructed to consult with their health care providers, when needed. If participants have questions regarding the content of the modules, they can submit their questions to the research staff, who will review the submitted questions and, if necessary, forward them to one of the experts on the expert panel comprising a selected team of professionals who have experience working with individuals with SCI and their family caregivers. The selected expert will provide additional resources or information to address the participants’ questions. The experts on the proposed project will not provide personal interventions to family caregivers; instead, they will focus on providing information to address the submitted questions only. Questions submitted by participants during the feasibility study will be used to modify and improve COMPANION. The research team will record participants’ submitted questions and the amount of time spent addressing each question and responding to participants. This information will also be used to further improve and modify COMPANION.

In the first 3 months of enrollment of participants in the intervention group in this feasibility RCT, the research staff remotely monitor online analytics (eg, login frequency, module progression) of COMPANION and contact a participant if no online activity is noted in a 2-week period in order to promote use and troubleshoot potential technical problems.

### Control Group

Control group participants will receive the usual care available at their local rehabilitation clinic. Immediately upon reaching the 6-month timepoint, control group participants will be given access to the current version of COMPANION.

### Feasibility Indicators

The RCT’s primary feasibility indicators will be used to address the COMPANION intervention’s design and fidelity by assessing process issues, resource issues, management issues, and treatment issues. See Table 1 for further information about the feasibility indicators.

**Table 1:** Feasibility indicators.

Feasibility indicator and components		Subindicator	Expectation
**Process**			
	Recruitment rate	Number of participants recruited, number of women and men recruited	Mean of 3-4 participants/month: total of 40
	Consent rate	Percentage of participants consenting	<10% participant refusal
	Retention rate	Percentage of participants with T3^a^ data	Complete data collection for >80%
	Perceived benefit	Responses to the SUS^b^, qualitative interviews at T3	>85% of responses strongly agree/agree, qualitative analysis informing clinical importance
	Assessor masking	Percentage unaware of group status	100% of participants not unmasking their treatment
**Resources**			
	Treatment adherence (COMPANION)	Completed all the modules	>85% of participants
	Data collection: participant and evaluator burden	Baseline, T2^c^, and T3	>85% of participants to complete in ≤2 hours, >85% of participants to complete in ≤1.5 hours
	Expert burden	Time (minutes) spent in answering participants’ questions and following up with them	Mean time spent per participant <2 hours for baseline and <1 hour for T2, <20% phone calls back for clarification
**Management**			
	Internet stability	Downtime due to technical or mechanical issues	>90% of participants not without internet for >2 days
	Participant processing time	Time from data collection to treatment	Mean time <10 days at each site
	Treatment administration issues	Posttreatment evaluation form (study educator)	Any issues identified modifiable without substantial changes to the protocol
**Treatment**			
	Dose level response	Correlation between training time and change score	A positive correlation

^a^T3: 6 months after baseline.

^b^SUS: System Usability Scale.

^c^T2: 3 months after baseline.

### Clinical Outcome Measures: Primary Outcome

Clinical outcomes will be measured via a digital survey administered at baseline, T2, and T3. The components of the survey and outcome measures are detailed next.

#### Subjective Burden

The primary clinical outcome (ie, caregiver burden) will be measured through the survey using the Zarit Burden Interview [35. Studies have demonstrated a direct association between the caregiver burden and the quality of life for family caregivers [19]. As family caregivers’ appraisal of their ability to cope with their family members’ health condition is a direct predictor of their burden [14], and COMPANION aims to provide the required education and information for fcSCI so that they perceive and experience less challenges in providing care, we have selected fcSCI’s subjective burden as our primary clinical outcome. The short 12-item version of the Zarit Burden Interview will be used to assess the subjective burden [35]. The fcSCI are asked to use a 5-point scale (from 0=never to 4=nearly always) to evaluate how often they experience certain feelings. Total scores can range from a minimum of 0 to a maximum of 48. A total score ranging between 0 and 10 indicates no-to-mild burden, 10-20 indicates a mild-to-moderate burden, and >20 indicates a high burden. This measure has been considered as valid and reliable (Cronbach α=.78) among fcSCI [36].

### Clinical Outcome Measures: Secondary Outcomes

The survey administered at baseline will collect information on participants’ age, sex, gender, social support, annual household income, living situation, and chronic health. The fcSCI will be asked to answer similar questions for individuals with SCI. In addition to demographic information, several secondary outcomes measures will be administered at multiple timepoints. The components of the secondary outcomes measures and their timepoints are detailed next.

#### Objective Burden

The survey will be used to administer several secondary clinical outcome measures. The first secondary outcome will be measured using the 38-item Dutch Objective Burden Inventory assessing fcSCI’s objective burden [37]. Each questionnaire item lists a specific caregiving activity that corresponds to 1 of the following 4 domains: personal care (eg, helping with eating and drinking), practical support (eg, buying groceries), motivational support (eg, motivating to quit or reduce smoking), and emotional support (eg, showing understanding). The fcSCI are asked to rate their perceived burden for each task over the past 3 months using a 3-point scale (1=not at all burdensome, 2=somewhat burdensome, 3=very burdensome). Total scores are calculated as the average of all individual answer scores, ranging from 1 to 3. A higher total score indicates a higher objective burden. Testing of the measure has affirmed adequate validity and reliability among Dutch and Canadian caregivers [38,39].

#### Distress

The second secondary outcome will be measured using a short version of the Depression, Anxiety and Stress Scale (DASS) assessing distress [40]. A short version of the complete 42-item DASS, the DASS-21 contains 21 items assessing depression, anxiety, and stress on 3 separate subscales comprising 7 items each. Participants will report the intensity of their symptoms over the past week using a 4-point Likert scale (from 0=never to 3=always). Separate total scores ranging from a minimum of 0 to a maximum of 42 will be calculated for each subscale. Severity levels for depression, anxiety, and stress can be interpreted based on recommended cutoff scores, with higher scores indicating more distress. Cutoff scores for the Depression subscale distinguish responses as normal (0-9), mild (10-13), moderate (14-20), severe (21-27), and extremely severe (≥28). Testing of the DASS-21 among family caregivers has shown adequate reliability (α coefficient=.97) [41].

#### Relationship Quality Satisfaction

The third secondary outcome will be measured using the Dyadic Adjustment Scale (DAS-32) [42]. The DAS-32 consists of 32 items measuring the level of relationship quality satisfaction among dyads. Items in the DAS-32 correspond to 4 dimensions, or subscales, for measuring relationship quality: dyadic consensus (consensus on matters of importance to marital functioning), dyadic satisfaction, dyadic cohesion (closeness experienced by the couple), and affective expression (demonstrations of affection and sex relations). Each item of the questionnaire corresponds to 1 of the 4 subscales, which consist of varying response scales, including ordinal, Likert, and dichotomous scales. Scores for each subscale are calculated and added together to produce a total score ranging from 0 to 151, with a higher total score indicating less distress and high adjustment. Psychometric testing of the measure has demonstrated good reliability for each subscale (α=.85 for dyadic consensus, .67 for dyadic cohesion, .76 for affective expression, and .82 for dyadic satisfaction) in the general population [42].

#### Health-Related Quality of Life

The fourth secondary outcome will be measured using the Veterans RAND 12-item Health Survey (VR-12) assessing fcSCI’s health-related quality of life [43]. The VR-12 includes 12 questions corresponding to 8 domains: general health perceptions, physical functioning, role limitations due to physical problems, role limitations due to emotional problems, social functioning, bodily pain, vitality, and mental health [44]. The 8 domains can be summarized into separate physical and mental component scores. The VR-12 scoring algorithm is used to score physical and mental components based on weights derived from a 1990 American population sample standard, resulting in variable minimum and maximum score values [45]. Higher physical and mental components scores indicate better health. Two further questions (ie, 14 in total), which do not contribute to the component summaries, ask about physical health and emotional problems compared to 1 year ago. Health state values (also known as utility scores), reflecting the preferences of the Canadian population, can also be generated from VR-12 data [46]. Though validation of the VR-12 has not been conducted in family caregiver populations, the VR-12 has demonstrated adequate convergent validity in other populations [47].

#### Competence

The fifth secondary outcome will be measured using the Caregiving Competence Scale (CCS) [48]. The CCS is used to measure a caregiver’s self-assessment of the adequacy of their performance in their role. The 4-item questionnaire scores responses using two 4-point scales, with response options ranging from 1=“not at all” to 4=“very much” in the first scale and from 1=“not at all” to 4=“very” in the second scale [48]. Score values range from 0 to 12, with higher scores indicating higher caregiving competence. The CCS has demonstrated adequate internal consistency (Cronbach α=.78-.82) in family caregiver research [49,50].

#### Usability

The sixth secondary outcome will be measured using the System Usability Scale (SUS) to capture the opinion of participants regarding the usability of COMPANION. Originally created by Brooke [51], the SUS consists of a 10-item questionnaire using a 5-point scale, where options range from strongly agree to strongly disagree. Raw scores range from 0 to 40 and are algorithmically converted into meaningful SUS scores ranging from 0 to 100. Higher scores indicate higher perceived usability of the program. Participants in the intervention group will complete this measure at T2 and T3.

#### Caregiver Costs

The final secondary outcome will be measured using the Caregiver Indirect and Informal Care Cost Assessment Questionnaire (CIIQ) to capture information estimating informal care costs [52]. The fcSCI will be asked to provide responses for 13 items with information including, but not limited to, work status, time missed at work, productivity, and physical and emotional caregiving duties. Calculations will be conducted using responses to items on the CIIQ to estimate fcSCI indirect and informal care costs. Total scores can range from as low as 0 to any maximum number and report cost in dollars. Higher scores indicate a greater financial impact due to informal care costs. The CIIQ will be coupled with a bespoke item asking about additional money the fcSCI spend on themselves. To minimize concerns about recall, a 1-week recall period will be used for the CIIQ and a 3-month recall period will be used for the bespoke item.

### Qualitative Interviews

When the feasibility RCT is complete, semistructured interviews (~45-60 minutes) will be used to explore fcSCI’s experiences in the intervention group with COMPANION. The option to take part in the semistructured interviews will be available to participants in the intervention group as soon as they complete T3 survey data collection. An interview guide has been created to capture multiple aspects of fcSCI’s experiences using COMPANION. As a complimentary method to assess usability, the interviews will also be used in combination with the SUS [51] and feasibility indicators to evaluate the usability of COMPANION. Participants will be asked to share their perspective on the advantages and disadvantages of COMPANION. A directed content analysis approach will be used to form initial coding categories from existing evidence and study hypotheses [53].

To promote the credibility and trustworthiness of qualitative data, we will use several methods. Field notes will be used as a self-reflective tool. Member checking will be used; that is, participants will be invited at the end of each interview to review the preliminary findings and provide feedback to determine how well they resonate with them. Multiple investigators with diverse backgrounds will be involved in collaborative theme analysis and data coding as a form of triangulation [54]. Interview results will be integrated with the quantitative results to provide a more in-depth assessment of benefits and user acceptability [55]. Peer debriefing will be used to further support triangulation. To ensure transferability of the qualitative data in our reports, we will follow the Consolidated Criteria for Reporting Qualitative Research (COREQ) report guidelines [56]. Specifically, we will provide detailed information about the setting, participants, and interviewers’ and research team’s backgrounds. We will also provide a detailed description of the data collection procedure, analyses of the qualitative data, and our procedure for developing codes and themes to ensure dependability of our method. Furthermore, we followed the CONSORT (Consolidated Standards of Reporting Trials) eHealth Form (see Multimedia Appendix 2) [57].

### Data Management

Participant consent forms and all data collection will be administered online via Qualtrics. Data will be stored on Qualtrics and will be accessible only to research personnel who are granted access by the research staff. Participants will receive a link to the consent form via email with instructions on how to complete the form. After signing the online consent form, each participant will schedule a video call meeting, during which they will complete baseline data collection measures with the help of the study manager. Immediately after the participant and study manager complete the baseline questionnaire, the study manager will leave the call and be replaced by the research staff, who will randomize the participant into the intervention or the control group using the secure online software [32]. The participants will be given instructions based on their group allocation and asked not to reveal their group to the study manager, who will remain masked to the randomization result. Participant IDs and group allocation will be recorded on a secure, password-protected digital tracking sheet. Only research personnel involved with the group allocation will have access to the tracking sheet, and the study manager will not be able to access any information on the sheet related to participant group allocation. At T2 and T3 timepoints, participants will be sent an email containing a unique link to the next Qualtrics questionnaire and will be asked to complete the survey on their own.

### Data Analysis

#### Statistical Analysis

All data analysis will be conducted by the research team independently from the study sponsor. Descriptive analysis will be used to describe our sample and consider clinical outcomes and study feasibility. The most current versions of R statistical software (R Foundation for Statistical Computing) [58] and IBM SPSS software [59] will be used. The 1-sample Kolmogorov-Smirnov test will be performed to evaluate data distribution. Demographic and outcome variables will be summarized by groups using means (SDs), frequencies, and proportions. Similar to previous feasibility studies [60], the means (SDs) calculated in the feasibility RCT will be used to estimate the effect size and the variance necessary for the full RCT. Focuses of the analysis will include investigating differences in primary and secondary effectiveness outcomes for the intervention and control groups at each timepoint. We will include an interaction between time and group for all models to account for the possibility of effect modification by time. Estimated marginal means will be used to conduct post hoc analyses, and pairwise comparisons of estimated marginal means will be conducted to examine specific differences between groups at each timepoint. Participant IDs will be used as a random effect. Multiple imputations will be used to deal with missing data. Furthermore, descriptive statistics will be used to assess participants’ online usage data of the eHealth program (eg, the amount of time participants spent accessing the modules) to evaluate feasibility indicators (dose level response, treatment adherence) related to resource and treatment issues [61].

#### Feasibility

Feasibility outcomes will be treated as binary based on the expectations, as shown in Table 1. Specific objectives will be evaluated as a “success,” indicating that no major adaptation of the protocol is needed before proceeding with a definitive RCT, or “revise,” indicating changes must be made before continuing with the definitive RCT.

### Qualitative Interviews

Interview transcripts will be examined using an inductive thematic analysis approach [62]. Data will be analyzed to identify themes and subthemes. Investigators will generate initial codes individually and then work with the entire team, including our caregiver partners, to identify themes, review and define themes, and select examples to support the themes [62]. The qualitative findings will be used to interpret the clinical outcomes, such as key intervention ingredients, relevant outcomes that might not be captured, and an explanation for potential conflicting quantitative results. Quantitative and qualitative findings for participants will be integrated as a form of triangulation and summarized to identify patterns and paradoxes between the results [63].

## Results

This research was funded by the Craig H Neilsen Foundation PSR Pilot Grant (PSR2-17) beginning in April 2022. After the first 2 study phases, during which COMPANION was codeveloped with caregiver partners, recruitment and data collection for the RCT began in June 2024 and is currently ongoing. We enrolled 54 participants at the end of baseline. Data collection was completed in April 2025. Data analysis for the RCT is expected to begin in June 2025. The fcSCI randomized into the intervention group have been given access to COMPANION after randomization.

## Discussion

### Summary

This study aims to investigate the feasibility of the COMPANION eHealth protocol. COMPANION e-Health modules have been developed to provide the education needed for family caregivers of individuals with SCI. By providing the necessary education, we aim to reduce the caregiver burden and improve caregivers’ overall well-being. Health education using online approaches has been lauded for being interactive and enabling learners to engage over sustained periods. Benefits of eHealth can include improving the quality of care [37,64,65], enhancing communication between health care users and providers [66], reducing costs [67], and increasing access to evidence-based health information. As the COMPANION modules were designed specifically to address aspects of caregiving that are significant to fcSCI, COMPANION has the potential to increase fcSCI’s knowledge and health literacy around challenges that they face when providing care (eg, emotional challenges, challenges in conducting medical tasks, challenges with isolation and accessing social support). By providing access to education and resources regarding factors that are known to mitigate the caregiver burden for fcSCI, such as social support [68] and coping appraisal [14], COMPANION may be used to impact fcSCI’s overall feelings of a burden, quality of life, and well-being. Findings from conducting the feasibility RCT that evaluate the feasibility and acceptability of our study procedures can be used to inform the design of a definitive RCT assessing the effectiveness and impact of COMPANION on clinical outcomes compared to usual care pursued by fcSCI. Conducting a definitive RCT can further contribute meaningfully to family caregiver research and literature about mitigating the burden experienced by caregivers of individuals with SCI.

Analysis has not yet begun for this study. We anticipate that a large percentage of fcSCI who are approached to participate in our study will be female due to our recruitment plan to ask Wives and Girlfriends of Spinal Cord Injury to distribute recruitment materials to their members, who are primarily female fcSCI. Though women have made up the majority of fcSCI participants in recent studies with similar populations [5,69,70], we plan to use recruitment methods aimed at diversifying the gender spectrum of the fcSCI we approach by additionally contacting participants through our network of previous patients and research participants of our center and distributing recruitment information through social media advertisements. Additionally, the literature suggests that eHealth approaches may attract a younger demographic of users who may be more experienced using online platforms than older populations, who may be discouraged from using the platforms or participating [21]. To support participants’ access and experience using COMPANION, participants will be sent written instructions on how to access and navigate COMPANION and its modules, encouraged to use COMPANION with their family members with SCI, and instructed to contact the research staff with any questions about using the program. We also plan to monitor intervention group participants’ online activity during the study and reach out to participants if a time span of 2 weeks passes without any activity recorded. This communication will give participants an opportunity to voice any barriers they experience when using COMPANION and receive technological or other guiding support from the research staff [23].

Our knowledge translation plan will target fcSCI, individuals with SCI, and clinicians across Canada and the United States, leveraging existing communication tools, such as websites (eg, health authorities in British Columbia [BC]) and electronic and print newsletters of local and national audiences (eg, ICORD, Spinal Cord Injury Research Evidence, Spinal Cord Injury BC). All team members, including our caregiver partners, will contribute to the presentations and manuscripts. We, along with our caregiver partners, will share plain language summaries with fcSCI via newsletters, social media, and websites.

## Appendix

Multimedia Appendix 1 SPIRIT schedule of enrollment, interventions, and assessments for the RCT. RCT: randomized controlled trial; SPIRIT Standard Protocol Items: Recommendations for Interventional Trials.

Multimedia Appendix 2 CONSORT eHEALTH checklist (V 1.6.1).

Multimedia Appendix 3 Peer review report from the Craig H. Neilsen Foundation PSR 2022 FGA Committee.

## Abbreviations

CCS: Caregiving Competence Scale
CIIQ: Caregiver Indirect and Informal Care Cost Assessment Questionnaire
CONSORT: Consolidated Standards of Reporting Trials
DAS-32: 32-item Dyadic Adjustment Scale
DASS: Depression, Anxiety and Stress Scale
fcSCI: family caregivers of individuals with spinal cord injury
ICORD: International Collaboration on Repair Discoveries
RCT: randomized controlled trial
SCI: spinal cord injury
SUS: System Usability Scale
T2: 3 months after baseline
T3: 6 months after baseline
VR-12: Veterans RAND 12-item Health Survey
