# Supplementary figures and images for: Codeveloping a Novel Intervention to Promote the Well-Being of Family Caregivers of Individuals With Spinal Cord Injury: Protocol for a Feasibility Randomized Control Trial
Source: JMIR Res Protoc. 2025 Sep 25;14:e67709. doi: 10.2196/67709 (PMC12511813; doi:10.2196/67709)

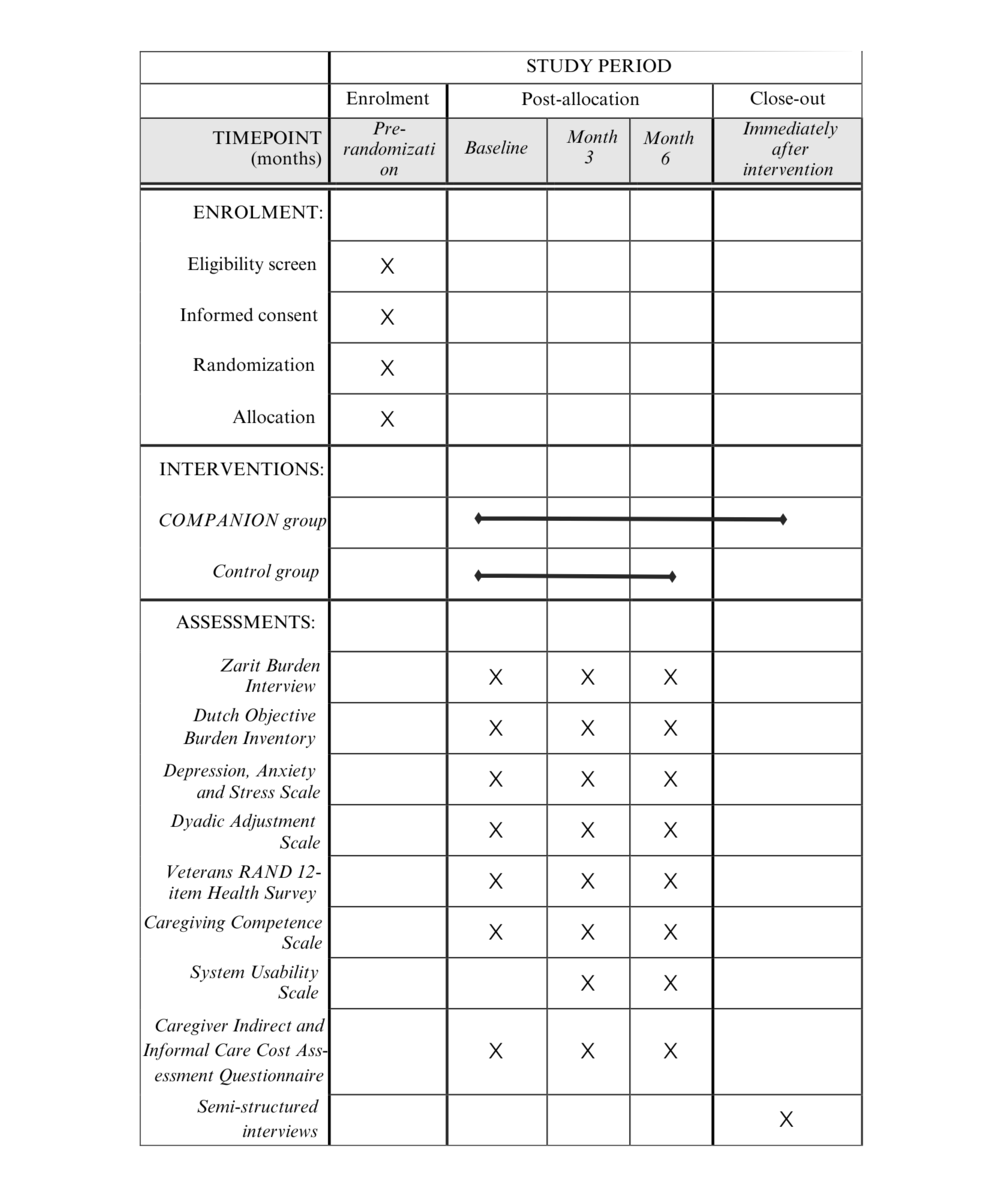

Supplement: Multimedia Appendix 1 [file resprot_v14i1e67709_app1.png]
