# Supplementary material for: Codeveloping a Novel Intervention to Promote the Well-Being of Family Caregivers of Individuals With Spinal Cord Injury: Protocol for a Feasibility Randomized Control Trial
Source: JMIR Res Protoc. 2025 Sep 25;14:e67709. doi: 10.2196/67709 (PMC12511813; doi:10.2196/67709)
Supplement: Multimedia Appendix 3 [file resprot_v14i1e67709_app3.pdf]

CRAIG·H NEILSEN FOUNDATION

**Applicant:**	Miller, Bill
**Title:**	Co-developing a novel intervention to promote wellbeing of family caregivers of patients with spinal cord injury (iCare)
**Program:**	PSR Pilot Grants (PSR2-17)
**Institution:**	University of British Columbia
**App #:**	865706

View Review Information

Close Window Print

Committee: PSR 2022 FGA

Summary Statement

Text Admin Summary:

The Neilsen Foundation has conditionally approved funding of this proposal. Reviewers expressed enthusiasm for the project's focus on caregiver burden and incorporating a health education approach based on adult learning principles. Though they commended the project for bringing approaches and investigators from other fields into SCI research, they stressed a need for stronger SCI expertise on the team. Reaching out to SCI experts at ICORD is highly recommended. Please see the reviewer critiques for more discussion of strengths and weaknesses.

Reviewer Role: Primary

OVERALL IMPACT

After considering all of the review criteria, summarize the significant strengths and weaknesses of the application. Will this project help establish a junior PI's research program and/or enhance an established program? Does the project have the potential for "high gain" to balance risk? Does the project scope suit the funding level and does it have the potential to exert a sustained powerful influence and/or be an important contribution to the field of SCI psychosocial research?

:

The proposed project will develop an eHealth caregiver education program, called iCare, based on adult learning principles.

The program is more in line with a health education approach to support, rather than a psychological intervention, per se. As such, its emphasis on the unique education and learning needs of the individual caregiver is an attractive feature, as iCare will have a digital, "person-to-person" capability. (These may be customized and interactive). The project will then conduct a feasibility RCT to determine if a larger RCT can be conducted. The PI does not meet most definitions of a "junior colleague."

SIGNIFICANCE
1. Does the project address an important problem or a critical barrier in the field?

2. If the Aims of the project are achieved, how would this work change or enhance current methods, technologies, treatments, services, or interventions?

:
The project will address a significant problem in SCI rehabilitation. There are no real, ongoing "treatment as usual" programs for family caregivers, and our health care systems are unlikely to develop and provide meaningful services under present conditions, as they are currently not financially sustainable. Programs that provide support, education and guidance at low cost to the caregiver (FC) are needed. If successful, the proposed project may offer an approach to helping FCs.

RELEVANCE
1. How is this project relevant to the mission of the Neilsen Foundation?

2. How is this project relevant to the goal of the PSR portfolio?

:
The proposed project is appropriate to the mission of the Neilsen Foundation and it is suited to the portfolio. Programs that assist FCs in the home are needed and this is a topic well within the scope of the portfolio.

INVESTIGATOR(S)
1. Are the PI, collaborators, and other contributors well suited to the project? Are relevant SCI and psychosocial research expertise represented on the proposed project team?

2. If the PI is a junior investigator, does he/she have appropriate experience, training and facilities to do the proposed work? If the PI is an established investigator, has he/she demonstrated an ongoing record of accomplishments that have advanced their field(s)?

:
The PI responded to previous feedback and asserts their research and clinical expertise in SCI rehabilitation. The PI's Google Scholar page lists 14 papers with "spinal cord" in the title, and one with "family caregiver" in the article title. Most of these studies do not concern interventions for FCs. However, the PI has a few papers reporting the use of digital technologies to provide rehabilitation-related services and activities (in the community and in the home). Most studies concern measurement and instrumentation. In general, the PI has a research record that supports the application, and the team appears to have the basic expertise to conduct a project of this nature. However, it is unclear why a "grief counselor" is included on the team (see budget justification). It is not clear why a grief counselor is needed to "advise the team on study process, recruitment, dissemination." This reviewer understands the person named for this role is a FC/spouse, but the budget justification and title does not correspond with the narrative. Further, the colleague who will "co-supervise" the iCare project (listed as "other" in the budget) is a health psychology and counselor with several papers concerning caregivers in chronic pain scenarios; none appear to be associated with SCI, or indicative of relevant clinical research concerning FC in SCI (based on the papers listed on their Google Scholar page). Consequently, issues and concerns about the quality of psychological expertise on the team persist, and these are critical matters, concerning the documented issues we know many FCs face in SCI.

INNOVATION

1. Does the project challenge and seek to shift current research or clinical practice/interventional paradigms by utilizing novel theoretical concepts, approaches or methodologies, instrumentation, or interventions?

2. And/or does this application apply concepts, approaches or methodologies, instrumentation, or interventions from another field of research to SCI?

3. And/or is a refinement, improvement, or new application of theoretical concepts, approaches or methodologies, instrumentation, or interventions proposed?

:

The health education approach is reasonable and the proposed project could potentially make a useful contribution to the field. The use of eHealth applications and other long-distance techniques appears to be the best available methods at present to help FCs in the home.

APPROACH

1. Is the project well-suited to the Pilot stage? Is the future applicability or next steps that rely on the Pilot data being sought discussed (i.e., does this research help lay the groundwork for future studies or describe plans for the dissemination and translation of knowledge that will impact current practices and approaches)?

2. Are the overall strategy, methodology, and analyses well-reasoned and appropriate to accomplish the specific Aims of the project, within the proposed project period and by the project team described? Are potential problems, alternative strategies, and benchmarks for success presented?

3. If the project is in the early stages of development, will the strategy establish feasibility and will particularly risky aspects be managed adequately?

4. If the project involves clinical and/or community-based research:
a. Does the project timeline include plans for study start-up time and sufficient subject recruitment?
b. Does the start-up plan adequately address regulatory approval, if required?
c. Will the National Institute of Neurological Disorders and Stroke, Common Data Elements be used? If not, is justification provided?

:

The proposed project has been streamlined to (a) develop the “outline” of the iCare program and refine it, and (b) conduct a feasibility RCT over 6 months with an N of 20 per arm. The control condition will receive “usual care”; it is not clear what this means (but they will be offered the iCare program upon completion). The outcome measures are reasonable although it does not appear that any of these are from the menu of instruments preferred by the foundation. It appears recruitment will focus on heterosexual couples (e.g., wagsofsci.com). Informed consent procedures are not explicated in the narrative. The randomization plan seems reasonable, as does the data analytic plan.

ENVIRONMENT

1. Will the institutional environment in which the work will be done contribute to the probability of success?

2. Are the institutional support, physical equipment and other resources available to the investigator(s) adequate for the project proposed?

3. Will the project benefit from unique features of their environment, subject populations, or collaborative arrangements?

:
The environment appears suitable and supportive of the project as described. The institutional setting appears to have a successful history in hosting funded projects.

TECHNICAL ELEMENTS
Your feedback on these items is valuable to us; however, the following components of the FGA should not alter the Overall Score of the application. Any relevant aspects of these elements should be included in written reviews, with the exception of Conflict of Interest (COI) concerns, which should be discussed privately with program staff.

1. Budget

2. Overlapping Funding

3. Ethics/Safety – protection of human subjects, including safety and data monitoring

4. Diversity, Equity, and Inclusion (DEI) Statement – In addition to providing the required institutional DEI statement, applicants are allowed to include additional information regarding their effort to promote DEI.

5. Other/Additional Comments for the Applicant

6. Resubmission – When reviewing a resubmission, evaluate the application as now presented, taking into consideration the responses to comments from the previous review group and changes made to the project, but basing your final score on the overall merits of the current proposal.

:
No response entered

Reviewer Role: Secondary

OVERALL IMPACT
After considering all of the review criteria, summarize the significant strengths and weaknesses of the application. Will this project help establish a junior PI's research program and/or enhance an established program? Does the project have the potential for "high gain" to balance risk? Does the project scope suit the funding level and does it have the potential to exert a sustained powerful influence and/or be an important contribution to the field of SCI psychosocial research?

:
The significant strengths of this application are a focus on an important problem (caregiver burden) in a modestly innovative manner, experienced investigators working in a resource-rich environment, and a design that is well matched to the Pilot funding mechanism. Weaknesses are minor and relate to omitting details about use of Common Data Elements, letters of support, and discussion of alternative procedures. The project appears to have the potential for "high gain" to balance risk by providing feasibility information for a future RCT.

SIGNIFICANCE
1. Does the project address an important problem or a critical barrier in the field?

2. If the Aims of the project are achieved, how would this work change or enhance current methods, technologies, treatments, services, or interventions?

:

This project addresses an important problem experienced by family caregivers of persons with SCI – the need for information to help them deal with the challenges they experience providing care. Providing the information and resources required and requested by family caregivers with an eHealth tool could help improve the physical and psychological wellbeing of family caregivers of individuals with SCI and, ultimately, to improve the well-being of the individuals with SCI.

RELEVANCE

1. How is this project relevant to the mission of the Neilsen Foundation?

2. How is this project relevant to the goal of the PSR portfolio?

:

This project is relevant to the Foundation's objectives to improve the quality of life for individuals with SCI and their family caregivers by empowering family caregivers and improving their psychological and physical wellbeing through education to care for themselves and for the individual with SCI. Indirectly, the investigators anticipate contributing to the wellbeing of individuals with SCI, as they rely on the support of their family caregivers for their daily activities. The project focuses on psychosocial issues relevant to the PSR portfolio, too.

INVESTIGATOR(S)

1. Are the PI, collaborators, and other contributors well suited to the project? Are relevant SCI and psychosocial research expertise represented on the proposed project team?

2. If the PI is a junior investigator, does he/she have appropriate experience, training and facilities to do the proposed work? If the PI is an established investigator, has he/she demonstrated an ongoing record of accomplishments that have advanced their field(s)?

:

The PI, collaborators, and other contributors are well suited to the project and possess the relevant SCI and psychosocial research expertise required for the proposed project. William Miller is an OT with a Ph.D. in epidemiology and biostatistics who is based at the GF Strong Rehabilitation Center, the largest rehabilitation center in British Columbia which has a unit for SCI. He is a Principal Investigator at ICORD, an interdisciplinary research center focused on spinal cord injury. His research focuses on interventions to address mobility disability and eHealth solutions in rehabilitation practice. Somayyeh Mohammadi, has a PhD in Health psychology and is a Registered Clinical Counsellor; she will co-lead the project with Dr. Miller, and focus on participant recruitment, data collection and training. She developed the eHealth module which will be used in this project. Julie Robillard is an expert in eHealth and patient experience, and is a Scientist in Patient Experience at BC Children's and Women's Hospital. She brings expertise in KT, health literacy, tool development and validation and in-patient engagement in research. David Whitehurst brings expertise in health economics and quality of life research, and experience in the design and conduct of economic evaluations.

INNOVATION

1. Does the project challenge and seek to shift current research or clinical practice/interventional paradigms by utilizing novel theoretical concepts, approaches or methodologies, instrumentation, or interventions?

2. And/or does this application apply concepts, approaches or methodologies, instrumentation, or interventions from another field of research to SCI?

3. And/or is a refinement, improvement, or new application of theoretical concepts, approaches or methodologies, instrumentation, or interventions proposed?

:

This application applies approaches, methodologies, and instrumentation from other fields of research to SCI: caregiver burden research, adult learning principles, and eHealth technology. The proposed iCare Program is an interactive eHealth education program that is based on adult learning principles and a user-centered approach. It features a digital interaction component with a counsellor, a physical/occupational therapist, and a nurse. If the aims are achieved, the project will provide an accessible, customized, effective and cost-efficient online education for family caregivers.

APPROACH

1. Is the project well-suited to the Pilot stage? Is the future applicability or next steps that rely on the Pilot data being sought discussed (i.e., does this research help lay the groundwork for future studies or describe plans for the dissemination and translation of knowledge that will impact current practices and approaches)?

2. Are the overall strategy, methodology, and analyses well-reasoned and appropriate to accomplish the specific Aims of the project, within the proposed project period and by the project team described? Are potential problems, alternative strategies, and benchmarks for success presented?

3. If the project is in the early stages of development, will the strategy establish feasibility and will particularly risky aspects be managed adequately?

4. If the project involves clinical and/or community-based research:
a. Does the project timeline include plans for study start-up time and sufficient subject recruitment?
b. Does the start-up plan adequately address regulatory approval, if required?
c. Will the National Institute of Neurological Disorders and Stroke, Common Data Elements be used? If not, is justification provided?

:

The project is well-suited to the Foundation's Pilot stage of funding. Results will provide data for a future RCT. The overall strategy, methodology, and analyses are well-reasoned and appropriate to accomplish the specific Aims of the project, within the proposed project period. The aims involve (1) co-developing the content of an eHealth program they name iCare which will build on preliminary work completed by their team, then (2) conducting a feasibility RCT to estimate the sample size and protocol for a future RCT. They plan to assess process, resource, management, and treatment issues in this Pilot study. They will engage an expert panel composed of a clinical psychologist, nurse, OT and PT with SCI experience, three family caregivers, and three individuals with SCI. They outline three sessions during which they will define and develop caregiving content. Aim 2 involves a two-arm intervention with 20 participants per arm. Assessments are scheduled at baseline, 3 and 6 months later. The primary outcome is subjective burden; secondary outcomes include caregivers' care activities, distress, relationship quality, health-related quality of life, system usability, and health care use. Qualitative interviews will provide supplemental feasibility information. Feasibility assessment is described well. The iCare intervention will consist of a comprehensive, structured library of material addressing medical care, financial and home aid, relationship issues, dealing with mental health issues, and learning life skills such as time management, problem-solving, and financial management. The investigators anticipate developing 10-12 interactive online modules that permit self-paced learning from home. The control arm will receive usual care and be offered access to iCare after completion of the study. The investigators do not explicitly describe potential problems and alternative strategies to achieve study benchmarks, though they have established relationships with the key individuals and organizations required for the success of this project. Investigators do not discuss use of NINDS Common Data Elements, though the selected instruments are reliable, valid, and relevant to the primary and secondary outcomes, and will not impose an excessive burden on participants.

ENVIRONMENT

1. Will the institutional environment in which the work will be done contribute to the probability of success?

2. Are the institutional support, physical equipment and other resources available to the investigator(s) adequate for the project proposed?

3. Will the project benefit from unique features of their environment, subject populations, or collaborative arrangements?

:

The institutional environment in which the work will be done should contribute to the probability of success. The institutional support, physical equipment and other resources available to the investigators are adequate for the project proposed. The project benefit from unique features of their environment, subject populations, and collaborative arrangements. The investigators have established partnerships with Wives and Girlfriends of Spinal Cord Injury, ICORD and GF Strong Rehabilitation Center which will enhance recruitment success. In Phase 3, they will collect data via Qualtrics in their institute. Dr. Miller's lab in the GF Strong Rehabilitation Center has the required analytic and data collection resources. The investigators provide no letters of support from the collaborating organizations.

TECHNICAL ELEMENTS

Your feedback on these items is valuable to us; however, the following components of the FGA should not alter the Overall Score of the application. Any relevant aspects of these elements should be included in written reviews, with the exception of Conflict of Interest (COI) concerns, which should be discussed privately with program staff.

1. Budget

2. Overlapping Funding

3. Ethics/Safety – protection of human subjects, including safety and data monitoring

4. Diversity, Equity, and Inclusion (DEI) Statement – In addition to providing the required institutional DEI statement, applicants are allowed to include additional information regarding their effort to promote DEI.

5. Other/Additional Comments for the Applicant

6. Resubmission – When reviewing a resubmission, evaluate the application as now presented, taking into consideration the responses to comments from the previous review group and changes made to the project, but basing your final score on the overall merits of the current proposal.

:

Resubmission – the applicant addresses the major concerns from the prior review and provides a response to each concern. They clarify that they have adopted the informal caregiving integrative model, obtained input from family caregivers of persons with SCI via semi-structured interviews, identified baseline levels of the primary outcome, clarify the investigators' effort, reduced the scope of work by completing interviews in preparation for this application, and justify the length of the outcome assessment.
